# Supplementary material for: Content-rich biological network constructed by mining PubMed abstracts
Source: BMC Bioinformatics. 2004 Oct 8;5:147. doi: 10.1186/1471-2105-5-147 (PMC528731; doi:10.1186/1471-2105-5-147)
Supplement: Additional File 2 — The original results of the above study (non-essential files are deleted to keep the file size under the limit set by BMC bioinformatics). [file 1471-2105-5-147-S2.bz2 › chilibotAdditionalFile2/dip05/153ID1423597E548/html/CDK4_CCND1.html]

 


 **CDK4** and **CCND1** 
  
Found 880 abstracts in PubMed, retrieved 05.  
 

 What does Google say? 
 PDF only 
| .edu only 

---

**Interactive relationship** (e.g. stimulation, inhibition, etc)

**Non-interactive relationship** (e.g. studied together, co-existance, homology, etc.)

- In a series of 23 surgical biopsies of grade II and III oligodendroglioma, cyclin D1  [ **CCND1** ] , E, A, B1,  **CDK4**  6, CDK2, Cdc2 and p27 Kip.1 have been studied by immunohistochemistry and Western blot.  Ref: 12873740 Neurosci Lett, 2003
- The restored ZD AZ forestomach epithelium displayed strong expression of Bax, a proapoptotic protein, and weak staining of cyclin D1  [ **CCND1** ]  and its catalytic partner  **Cdk4** , key regulatory proteins controlling G 1 to S progression.  Ref: 12873989 Cancer Res, 2003
- In contrast, proliferative ZD Wt forestomach showed strong expression of Bcl  [ **CCND1** ]  2, an antiapoptotic protein, and overexpression of cyclin D1  [ **CCND1** ]   **Cdk4** .  Ref: 12873989 Cancer Res, 2003
- Arsenic increased expression of the P21 protein and decreased levels of cyclin A, cyclin B1 and cyclin D1  [ **CCND1** ] , but expression of CDK2,  **CDK4** , CDK6, and cyclin E were not affected.  Ref: 12883267 Pancreas, 2003
- The high rate of cell proliferation was accompanied by overexpression of cell cycle progression and tumorigenesis biomarkers, including proliferating cell nuclear antigen, cyclin D1  [ **CCND1** ] , cyclin dependent kinase 4  [ **CDK4** ] , p53, cytokeratin 14, epidermal growth factor receptor, and by a reduced rate of apoptosis.  Ref: 12874033 Cancer ResCancer Res, 2003
- EGF administration after PH also significantly affected the activity of the cyclin D1  [ **CCND1** ]   **cdk4**  and cyclin E cdk2 complexes, mainly by changing their time progression it accelerated the increase in activity to 18h and caused a subsequent drop in activity after 34h.  Ref: 12876382 Cell Physiol Biochem, 2003
